# Elusive mustelids—18 months in the search of near‐threatened stoat (*Mustela erminea*) and weasel (*M. nivalis*) reveals low captures

**DOI:** 10.1002/ece3.11374

**Published:** 2024-05-01

**Authors:** Sofie Nørgaard Konradsen, Linnea Worsøe Havmøller, Charlotte Krag, Peter Rask Møller, Rasmus Worsøe Havmøller

**Affiliations:** ^1^ Department of Zoology, Natural History Museum of Denmark University of Copenhagen Copenhagen Denmark; ^2^ Norwegian College of Fishery Science UiT—The Arctic University of Norway Tromsø Norway; ^3^ Department for the Ecology of Animal Societies Max Planck Institute for Animal Behaviour Germany

**Keywords:** camera trapping, detection, individual identification, Mustelidae, wildlife monitoring

## Abstract

Stoat (*Mustela erminea*) and weasel (*M. nivalis)* are hard to monitor as they are elusive of nature and leave few identifying marks in their surroundings. Stoat and weasel are both fully protected in Denmark and are thought to be widely distributed throughout the country. Despite this stoat and weasel were listed on the Danish Red List as Near Threatened in 2019, as their densities and population trends are unknown. Using a modified novel camera trapping device, the Double‐Mostela, a wooden box comprising a tracking tunnel and two camera traps, we attempted to obtain density estimates based on identification of individual stoats and weasels. We deployed camera traps both inside Double‐Mostela traps and externally in three different study areas in northern Zealand, Denmark, and tested commercial, American scent‐based lures to attract stoat and weasel. We obtained very low seasonal trapping rates of weasel in two study areas, but in one study area, we obtained a seasonal trapping rate of stoat larger compared to another study using the Mostela. In one study area, both species were absent. We observed no effect of scent‐based lures in attracting small mustelids compared to non‐bait traps. Potential reasons behind low capture rates of weasel and stoat are suboptimal habitat placement and timing of deployment of the Double‐Mostelas, land‐use changes over the last 200 years, predation from larger predators, as well as unintended secondary poisoning with rodenticides. Due to the scarcity of weasel and stoat captures, we were unable to make density estimates based on identification of individuals; however, we identified potential features that could be used for identification and density estimates with more captures.

## Graphical abstract

The Double‐Mostela, a novel camera trapping device, allows for non‐invasive, double‐sided photos of stoats and weasels. We successfully captured both stoat and weasel, however, at very low trapping rates. We identified distinct features on the photos of stoat, allowing for density estimates to be made with more captures.

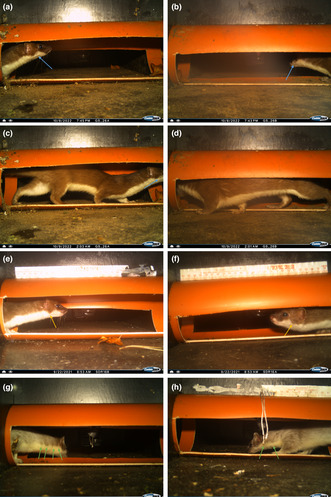

## 1 INTRODUCTION

Weasel (*Mustela nivalis*) and stoat (*M. erminea*) are highly specialized rodent predators with a widespread distribution (King & Powell, 2007). In parts of their distributional range, their population sizes seem to be decreasing (Hellstedt et al., 2006; Torre et al., 2018), and they have been given protection in, for example, Switzerland and Ireland due to the small and decreasing population sizes (Akdesir et al., 2018; Croose et al., 2021). Weasels and stoats are elusive and secretive of nature and leave few obvious natural marks, making them hard to study directly in the field (King & Powell, 2007). Because of this, monitoring and studying them have been conducted using various indirect methods instead. Traditionally, this has been done through live and kill trapping, as well as tracking of footprints and use of hair tubes (King & Edgar, 1977). Data from kill traps and hunting records of weasels and stoats have been used as indications of population densities (Hansen et al., 2007). However, decreasing records from these methods may not in themselves be a result of a decrease in population size but can also be related to decreasing trap effort (McDonald & Harris, 1999). Live trapping, and with this capture–mark–recapture or radiotracking, has been used to obtain density and home range estimates (Jedrzejewski et al., 1995; Zub et al., 2008). The use of hair tubes and tracking tunnels is quite effective for monitoring stoats and weasels as they both use rodent tunnels for hunting (García & Mateos, 2009; Graham, 2002; King & Powell, 2007; McAney, 2010). However, these methods can be expensive, time‐consuming, and require a high effort (García & Mateos, 2009). Prints in snow are limited to certain times of year, and footprints from small stoats and large weasels can overlap in size making the distinction between the species difficult (King & Powell, 2007).

Over the last few years, new and non‐invasive methods to monitor small mustelids have risen using camera traps. Camera trapping is a well‐known method used to monitor larger predators, as they can be set up and monitored without interference from researchers (Moruzzi et al., 2002). The traditional camera trap setup, however, may not efficiently capture small, fast‐moving species such as the weasel, and a team of Dutch researchers, therefore, developed a new method, named the Mostela, to monitor them (Mos & Hofmeester, 2020). The Mostela takes advantage of the natural tendency of weasels and stoats to explore holes. It consists of a wooden box with a camera trap and a tracking tunnel (see Methods for full description) (Mos & Hofmeester, 2020). The Mostela has been successfully used to capture mainly weasels but also stoats in England, Ireland, and the Netherlands (Croose et al., 2021; Croose & Carter, 2019; Mos & Hofmeester, 2020), and photos obtained have been used for identification of individual weasels (Mos & Hofmeester, 2020). However, stoats do not always enter traps and tunnels even if they are known to be present in an area (Brown, 2001; Croose & Carter, 2019; Dilks & Lawrence, 2000). To overcome this, Croose et al. (2021) also deployed external cameras directed toward the Mostela, capturing stoats investigating the box but not entering it. In the Netherlands, the Mostela was successful in detecting weasels without the use of bait or lure, however, scent‐based lures or baits are commonly used when monitoring mustelids (Garvey et al., 2017; Jedrzejewski et al., 1995; Kupferman et al., 2021; Macdonald et al., 2004). Commercial, American scent‐based lures have been used to attract pine marten (*Martes martes*) in Switzerland with an increase in detectability (Burki et al., 2010), whereas there was no effect of lures used to attract stoats and weasels in England (Croose & Carter, 2019).

Weasel was given full protection in Denmark in 1967, and as a result of decreasing captures during hunting efforts, stoat was also protected in 1982 (Elmeros et al., 2007a). With the revision of the Danish “Red List” in 2019, both species were given the status of “Near Threatened” (Elmeros & Sunde, 2019a, 2019b), but data supporting the presumed population decline are lacking. In 2005, the Danish Mammal Atlas Project mapped the distribution of all mammals in Denmark using different monitoring techniques (Hansen et al., 2007). Records of both stoat and weasel were based on random observations mainly through dead animals delivered to conservators or museums (Elmeros et al., 2007a, 2007b). Based on the Danish Mammal Atlas Project, stoats and weasels are thought to be distributed throughout Denmark, but there are no population density estimates. However, over the last few years, reports of sightings have sharply declined, and The Natural History Museum of Denmark is receiving dramatically fewer dead stoats and weasels (personal communication, Daniel Klingberg Johannsson). This information points to the presumed population decline in both species which led to the status of “Near Threatened.”

In the present study, we aimed to estimate the density of stoats and weasels in three different study areas in Denmark using the novel camera trapping device, the Double‐Mostela. We modified the original Mostela by adding an extra camera trap to capture animals from both sides in the hope of identifying individual weasels and stoats. Identification of individual weasels based on individual markings of the fur has been done before (Mos & Hofmeester, 2020), however, to the best of our knowledge a similar approach has not been tested on stoats. Inspired by Croose et al. (2021), we added an external camera facing the Double‐Mostela boxes in one of the study areas to capture potential stoats and weasels visiting a camera deployment but not entering the Double‐Mostela. We recorded habitat types at each deployment site to investigate preferences of stoats and weasels. We also evaluated the effect of using commercial, American scent‐based lures, traditionally used for hunting, to attract European mustelids.

## 2 METHODS AND MATERIALS

### 2.1 Study areas

Unlike other study sites investigated using the Mostela (Croose et al., 2021; Mos & Hofmeester, 2020), the Danish cultural landscape is sparse on hedgerows, stone walls, and other linear features. We, therefore, decided to investigate sites differing in size and degree of anthropogenic disturbance. Camera traps were set up in three different study areas (Overby Lyng, Strødam Nature Reserve, and Gribskov) in northern Zealand, Denmark. The region has mild winters and cold summers with an average annual temperature of 9.1°C (DMI, 2021b), and an average annual precipitation of 782 mm (DMI, 2021a).

**FIGURE 1:**
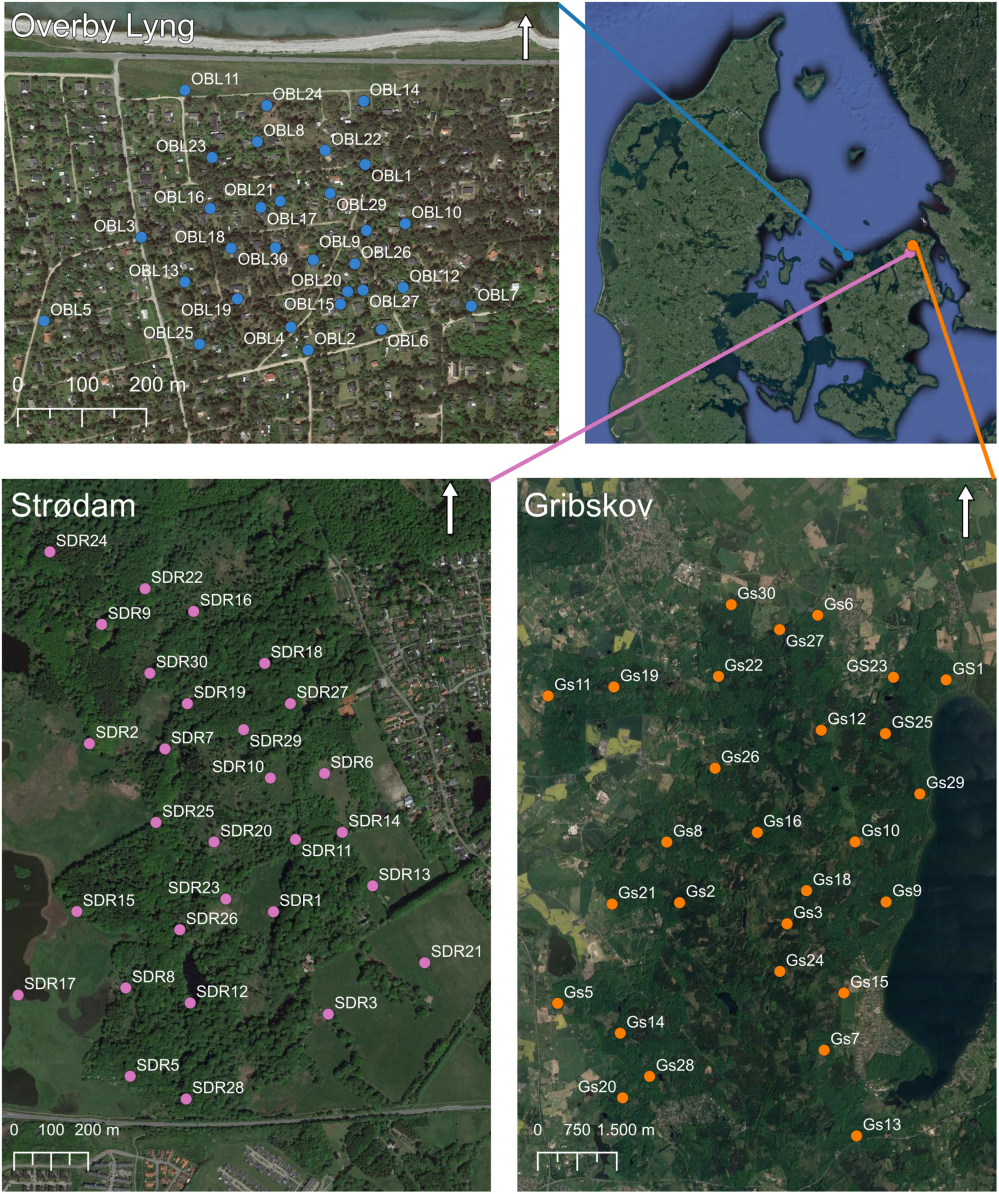
The three study areas, Overby Lyng (top left), Strødam (bottom left), and Gribskov (bottom right), and the placement of the Double‐Mostelas as well as external cameras in Gribskov. Note the different scales on the maps.

Overby Lyng (55°57′31.89″ N, 11°26′33.00″ E) (Figure 1) is a summerhouse area with vegetation dominated by conifers and heaths (*Erica* spp.). Human disturbance is expected to be high in this study site.

Strødam Nature Reserve (55°57′35.43″ N, 12°16′24.56″ E) is located in the south‐western corner of the forest Gribskov (Figure 1). Strødam is approximately 160 ha of which 100 ha is forest (DN, 2023). Most of the forest has been closed off to the public and untouched since its protection in 1925. This makes it one of the longest untouched reserves in Denmark with multiple habitats and an expected low anthropogenic disturbance (DN, 2023). Part of the forest as well as an adjoining meadow are currently grazed by horses, cows, and sheep. The adjoining meadow is considered a part of the Strødam study area, even though it is not part of the Strødam Nature Reserve.

Gribskov (55°59′58.79″ N, 12°17′5.46″ E) is the fourth largest forest in Denmark with an approximate size of 5800 ha (Naturstyrelsen, 2014). The eastern part of Gribskov is bordered by Lake Esrum, and in the southwestern corner, it is connected to the study area Strødam (Figure 1). Today, about 25% of the area consists of Norway spruce (*Picea abies*), however, it has been decided that much of the conifer plantation is to be replaced by broad‐leaved trees native to the area (Naturstyrelsen, 2014). Gribskov is one of the largest, coherent natural areas in Denmark, but with a high visitor number, and therefore human disturbance is expected to be high throughout the forest.

### 2.2 The Double‐Mostela

**FIGURE 2:**
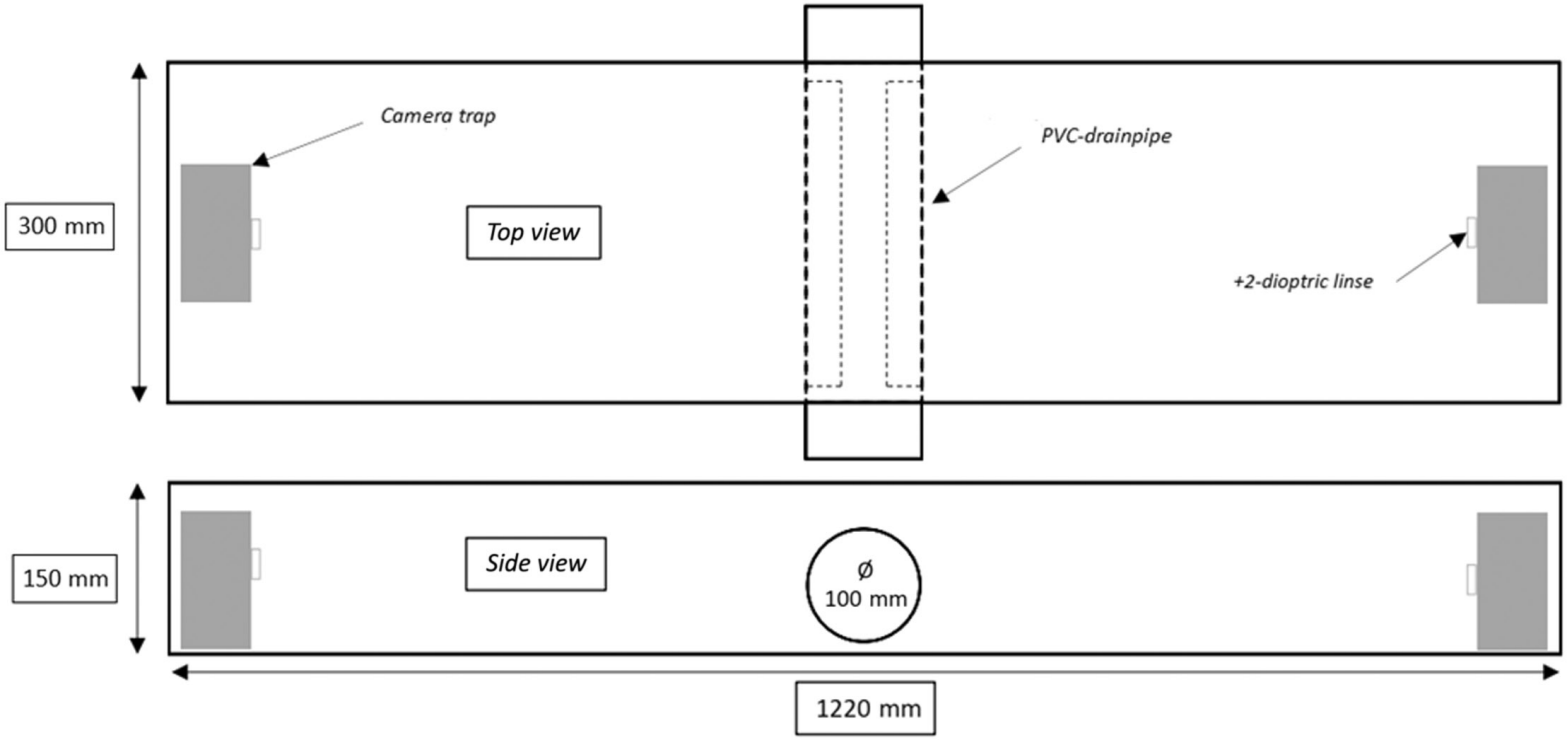
The modified Double‐Mostela used for this study. The box measures 1220 mm × 300 mm × 150 mm, and the PVC drainpipe has a diameter of 100 mm. Each camera has a + 2‐dioptric lens added in front of it to compensate for focus adjustment. The xenon flash was dimmed using gray duct tape.

In all three study areas, we monitored stoats and weasels using modified Mostela boxes after Mos and Hofmeester (2020). The Mostela is a wooden box specifically designed for small mustelids (Mos & Hofmeester, 2020) with a camera trap and a cut‐through PVC drainpipe inside it (Figure 2). Weasels and stoats have a natural tendency to explore tunnels (King & Powell, 2007), and the PVC drainpipe functions as a tracking tunnel to make them enter the Mostela, thereby triggering the camera (Mos & Hofmeester, 2020). The drainpipe we used had a diameter of 100 mm as Mos and Hofmeester (2020) found this to be the optimal diameter for weasel captures. For this project, we modified the Mostela to have double the length (measuring 1220 mm × 300 mm × 150 mm) and added an extra camera in the box (Figure 2). Our “Double‐Mostela” allows for captures of animals from both sides as they run through the PVC drainpipe situated in the middle of the Double‐Mostela. Getting captures from both sides of the small mustelids was chosen to make individual identification easier. Cameras (Cuddeback G‐5048, Cuddeback Non Typical Inc., Istanti, WI, USA) were set to take one photo when triggered using a xenon flash, and if triggered again, to take a photo as fast as possible after the first photo. We added a + 2‐dioptric lens from reading glasses in front of each camera to compensate for focus adjustment and dimmed the xenon flash with gray duct tape. The flaps on the outside of the camera's detection sensor were pulled down to maximize the detection zone. This was to ensure that the detection zone covered the entire width inside the Double‐Mostela.

### 2.3 Camera trapping

Camera trap grids were set up within different sampling areas in an attempt to explore potential differences in home range sizes, sampling of different habitats, and covering a full forest block. In all study areas, the Double‐Mostelas were placed to blend into the surroundings by placing them under fallen trunks or putting stones and branches on top of them.

Thirty Double‐Mostelas were deployed in Overby Lyng from June 10, 2021, until July 11, 2021 (Table 1). They were set up in thickets and covered a total area of 0.4 km^2^ (Figure 1).

**TABLE 1:** Overview of deployment periods in the study areas Overby Lyng, Strødam, and Gribskov.

Study area	Deployment period	Camera method	No. camera trap sites	Camera trap days	Independent animal captures
Overby Lyng	Jun. to Jul. 2021	Double‐Mostela	30	930	186
Strødam	Sep. 2021 to Sep. 2022	Double‐Mostela	29^a^	10,744	32,686
Gribskov	Oct. 2022 to Jan. 2023	Double‐Mostela	28^a^	3248	15,125
Gribskov	Oct. 2022 to Jan. 2023	External	28	1130	173
Strødam	Sep. 2022 to Jan. 2023	External	9	1134	413

*Note*: Camera method is either Double‐Mostelas or external cameras. External cameras in Gribskov deployed simultaneously as the Double‐Mostelas and facing them. External cameras in Strødam deployed separately during test of commercial lures.

^a^ Number of deployed Double‐Mostelas decrease, as the two Double‐Mostelas were destroyed by grazing livestock present at the deployment site.

In Strødam, we set up 29 Double‐Mostelas from September 09, 2021, until September 20, 2022 (Table 1). They covered a total area of about 1.5 km^2^ (Figure 1) and were set up with an average distance between Double‐Mostelas of 225 m. The Double‐Mostelas were set up in six different habitat types in Strødam (broadleaf forest, conifer forest, open area dominated by grasses, hedgerows, forest clearing, and wet, defined as Double‐Mostelas set up within 1 meter of water with soils that are consistently moist throughout the year). At the end of the deployment period in Strødam, we tested four different commercial American scent‐based lures to see if these would attract smaller mustelids to the Double‐Mostelas (Table S1). Lure was applied to a small 3D‐printed, plastic container and hung up inside the PVC drainpipe. When switching between lures, the old container was discarded and the lure was applied to a new one.

**FIGURE 3:**
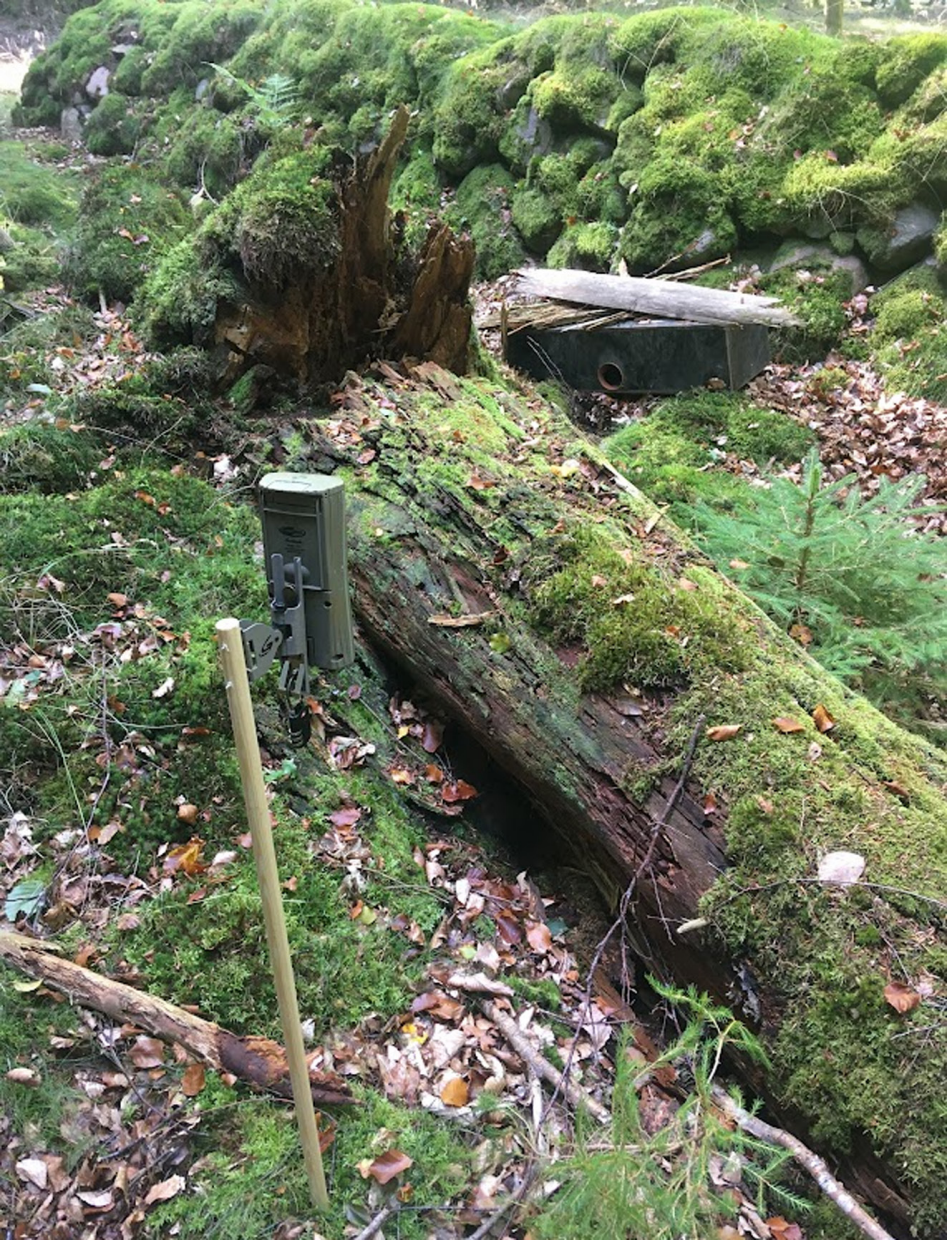
Setup of the Double‐Mostela and the external camera in Gribskov.

In Gribskov, we set up 28 Double‐Mostelas on October 03, 2022. Photos were retrieved from the cameras in Gribskov on January 26, 2023 (Table 1). The total area covered by the Double‐Mostelas is larger in Gribskov (approximately 50 km^2^) than in the other two study areas, as we focused on distributing the cameras in the entire forest (Figure 1). In Gribskov, Double‐Mostelas were set up in four different habitat types (broadleaf forest, conifer forest, open area dominated by grasses, and wet, defined as Double‐Mostelas set up within 1 meter of water with soils that are consistently moist throughout the year), and focused on linear features in the landscape, as stoats and weasels are known to use these (King & Powell, 2007). We added an external camera facing each of the Double‐Mostelas at a distance of 3 m in Gribskov (Figure 3) to capture potential small mustelids investigating the Double‐Mostela but not entering it (Croose et al., 2021). We deployed Cuddeback Ambush cameras (Cuddeback Non Typical Inc., Istanti, WI, USA) with a xenon flash. The external camera was set to take one photo and one 10‐s video as fast as possible after each other. Due to the use of xenon flash, the external cameras can only take videos during daylight hours.

**FIGURE 4:**
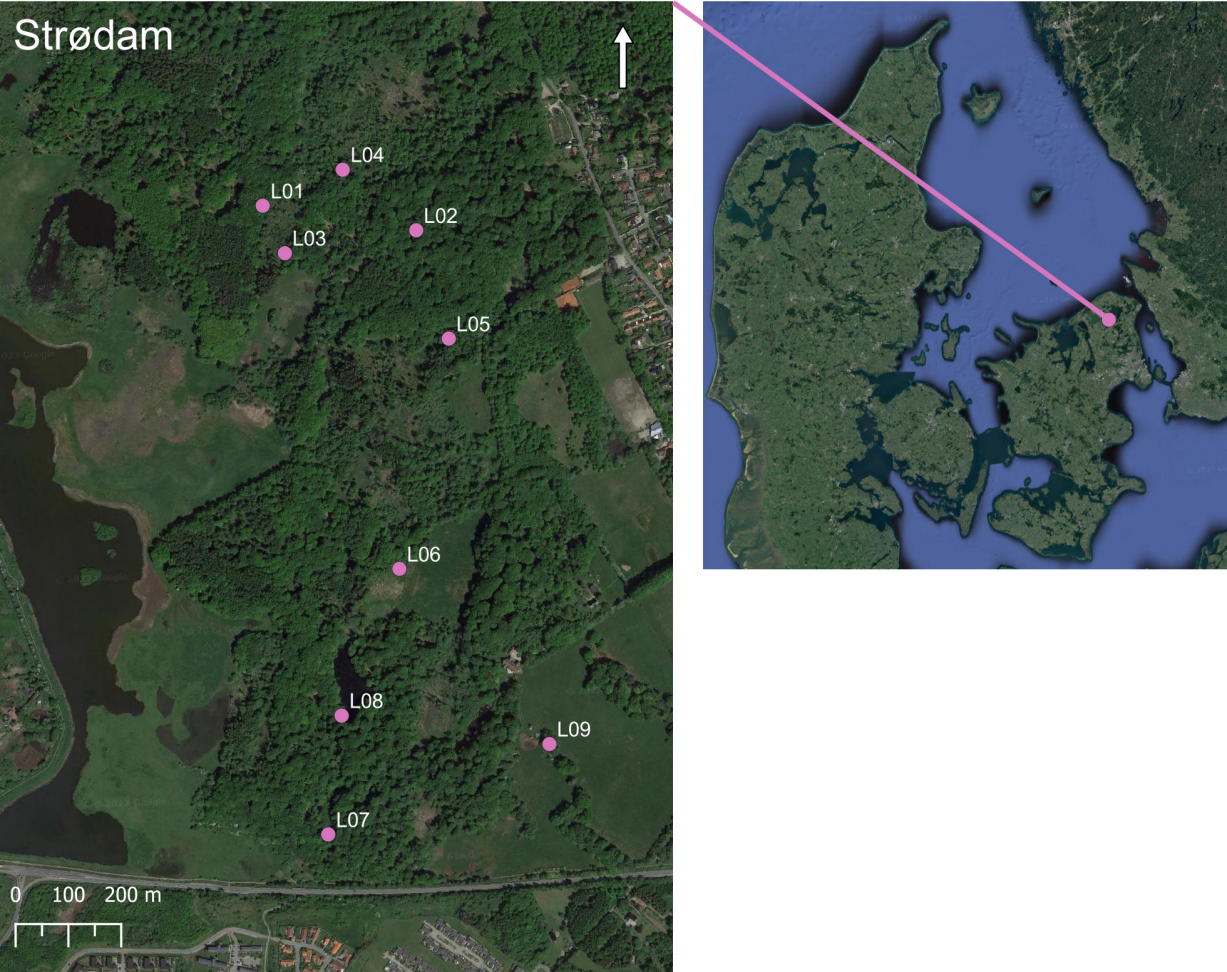
Camera trap sites for testing the effect of different scent‐based lures in Strødam.

**FIGURE 5:**
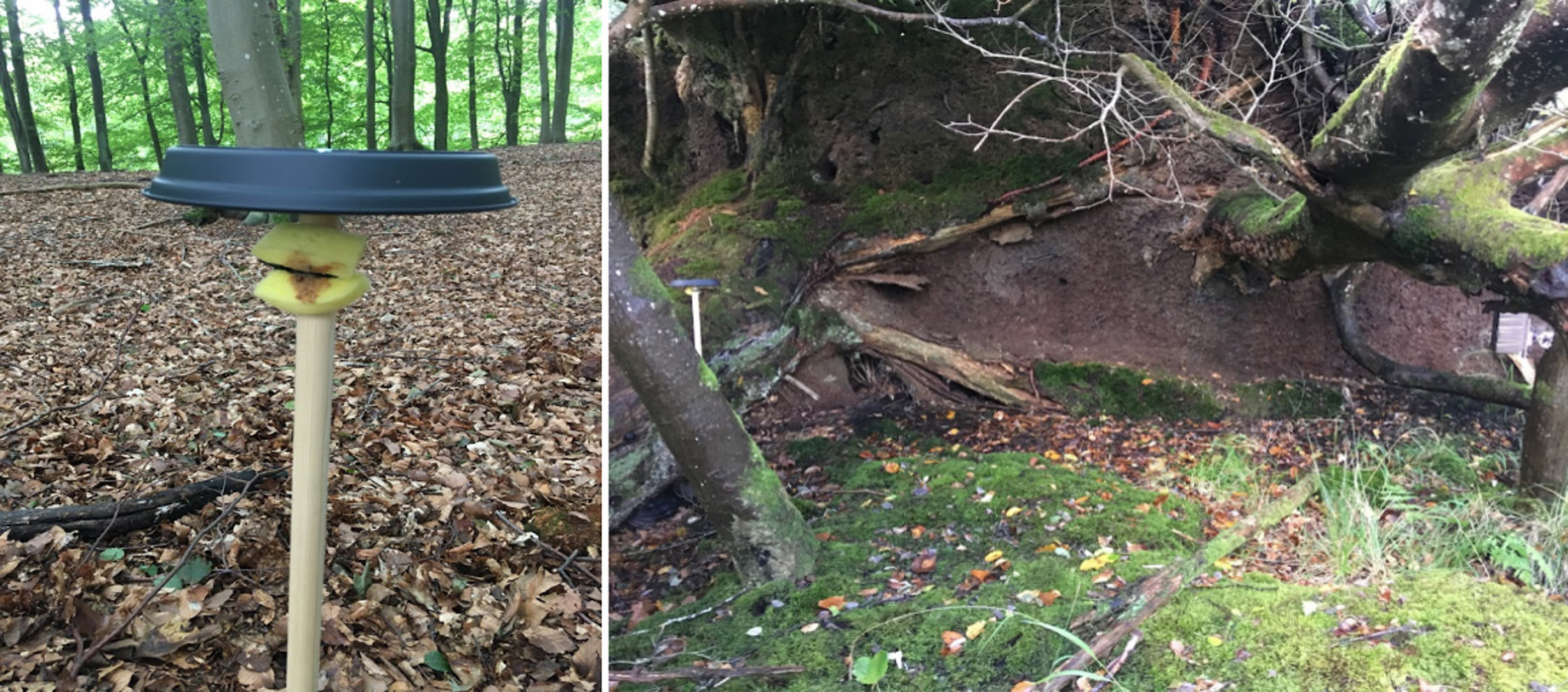
The setup of camera traps directed toward a wooden stake with a sponge zip‐tied under a cap on which the scent‐based lure was applied.

After the deployment of Double‐Mostelas, eight different scent‐based lures were tested separately in Strødam from September 19, 2022, until January 23, 2023 (for a full overview of deployment of the different lures, see Table 2). Cameras were placed in nine locations throughout Strødam Nature Reserve based on where we had captured stoats and weasels during the Double‐Mostela deployment (Figure 4). We focused on covering many different habitat types including beech forest floor, fallen trees, and proximity to open and wet areas. Lure was applied to a sponge zip‐tied to a wooden stake which the camera was facing (Figure 5). A plastic plate was screwed to the top of the stake to prevent the lure from being washed out of the sponge. The camera was deployed at a distance of 3 meters from the lure and positioned 0.3 m above the ground. The eight lures and one control circulated the camera trap sites with a 2‐week deployment period at each site. When rotating the lures, the sponge containing the old treatment was discarded, and a new lure was added to a clean sponge. The sponge was rubbed on the ground for the control. We used infrared Cuddeback G‐5017 (Cuddeback Non Typical Inc., Istanti, WI, USA) and set them to take three photos and one 10‐s video as fast as possible after each other when triggered.

**TABLE 2:** Deployment of the different scent‐based lures used in Strødam at each site, listed with start dates of the 2‐week deployment periods.

Start date	September 19, 2022	October 03, 2022	October 17, 2022	October 31, 2022	November 14, 2022	November 28, 2022	December 12, 2022	December 26, 2022	January 09, 2023
Site									
L01	Shellfish oil^1^	Hawbaker's^2^	Weasel supreme^3^	Mega musk^4^	Marten super all call^5^	Gusto^6^	Control	Salmon oil^7^	Weasel super all call^8^
L02	Hawbaker's	Weasel supreme	Mega musk	Marten super all call	Gusto	Control	Salmon oil	Weasel super all call	Shellfish oil
L03	Weasel Supreme	Mega musk	Marten super all call	Gusto	Control	Salmon oil	Weasel super all call	Shellfish oil	Hawbaker's
L04	Mega musk	Marten super all call	Gusto	Control	Salmon oil	Weasel super all call	Shellfish oil	Hawbaker's	Weasel supreme
L05	Marten super all call	Gusto	Control	Salmon oil	Weasel super all call	Shellfish oil	Hawbaker's	Weasel supreme	Mega musk
L06	Gusto	Control	Salmon oil	Weasel super all call	Shellfish oil	Hawbaker's	Weasel supreme	Mega musk	Marten super all call
L07	Control	Salmon oil	Weasel super all call	Shellfish oil	Hawbaker's	Weasel supreme	Mega musk	Marten super all call	Gusto
L08	Salmon oil	Weasel super all call	Shellfish oil	Hawbaker's	Weasel supreme	Mega musk	Marten super all call	Gusto	Control
L09	Weasel super all call	Shellfish oil	Hawbaker's	Weasel supreme	Mega musk	Marten super all call	Gusto	Control	Salmon oil

*Note*: Full names of scent‐based lures used: ^1^Shellfish Oil, ^2^Hawbaker's Weasel Lure, and ^3^Weasel Supreme—Reuwsaat's, ^4^Mega Musk—Carmen's, ^5^Marten Super All Call—Lenon's, ^6^Gusto, Long Distance Call Lure, and Predator Lure—Caven's, ^7^Sun Rendered Salmon Oil—Pint, ^8^Weasel Super All Call—Lenon's.

### 2.4 Data analyses

The open‐access software Wild.ID v. 1.0.1 (San Diego Supercomputer Center & Wildlife Insights, 2019) was used to annotate photos from the camera traps, both from the Double‐Mostelas and the external cameras. We identified to species level, if possible, alternatively to genus level. If this was not possible, the photos were annotated as “Unidentifiable.” All photos of mustelids were identified to species level. If photos did not contain animal captures, they were annotated as “Blank,” “Misfired,” or “Setup/Pickup” if taken during our work with deployment. For the Double‐Mostelas, we only annotated photos from the camera trap with most photos on it but retrieved the photos of stoats and weasels from the corresponding camera traps.

All data analyses were performed using the statistical program R Studio 2023.03.0 Build 386 (RStudio, 2020). An independent event was defined as a group of photos of the same species taken within 60 s of each other (Parsons et al., 2022). We performed a Kruskal–Wallis H test to test for differences between the lure treatments used in Strødam. We divided our data into four seasons: winter (December 22–March 21), spring (March 22–June 21), summer (June 22–September 21), and autumn (September 22–December 21). In Gribskov, autumn is defined as October 3–December 21 due to the deployment period starting there. We calculated seasonal trapping rates per 100 camera trap days for stoats and weasels. We identified the seasonal naive occupancy of stoats and weasels in each study area. We estimated detection and occupancy probability for stoats and weasels using the R package “unmarked” (Fiske & Chandler, 2011; Kellner et al., 2023). We used a single‐season occupancy model and defined an occasion as 1 day. We created our detection history binomially based on detection/non‐detection (1 = detection and 0 = non‐detection). We noted the habitat type, percent forest cover, and distance to water for every camera trap placement as site covariates for detection and occupancy probability. We measured the distance to the nearest occurrence of freshwater from each Double‐Mostela using the app EcoGIS Version 1.0 (6084) developed by Stewart MacDonald. We found forest cover at each site by sampling our site points with a GIS layer containing information on percent tree cover at a 30 × 30 m resolution (Hansen et al., 2013). However, the number of observations was insufficient to run models with site‐level covariates.

Maps of study areas with camera trap sites were generated using QGIS 3.22.3‐Białowieża (QGIS.org, 2023).

## 3 RESULTS

### 3.1 Double‐Mostela captures

We obtained a total number of 115,189 photos during 14,922 camera trap days for all three study areas combined. Deployment length was 930 camera trap days in Overby Lyng, 10,744 camera trap days in Strødam, and 3248 camera trap days in Gribskov (Table 1). Animal captures accounted for 99,034 of the photos, and of these 5 were of weasels and 67 were of stoats (Table S2). The number of independent 60‐s detections was 3 for weasels and 29 for stoats (Table 3). Seasonal trapping rates of stoats per 100 camera trap days using independent data were 0.12 (autumn) and 0.40 (winter) in Strødam and 0.71 (autumn) in Gribskov (Table 4). For weasels, seasonal trapping rates were 0.04 (autumn) and 0.04 (spring) in Strødam and 0.04 (autumn) in Gribskov (Table 4). Including stoats and weasels, a total of 40 species were captured using the Double‐Mostela (Table S2). From the Mustelidae family, the following species were captured: European pine marten (*Martes martes*), European badger (*Meles meles*), European polecat (*Mustela putorius*), and the introduced American mink (*Neovison vison*) (Table 3).

**TABLE 3:** Total and 60‐s independent captures of species from Mustelidae inside the Double‐Mostela boxes.

Scientific name	Vernacular name	Danish “Red List”	Study area	Total captures	Independent captures
*Martes foina* (Erxleben, 1777)	Beech marten	NT	O	4	2
*Martes martes* (Linnaeus, 1758)	European pine marten	NT	G	2	1
*Meles meles* (Linnaeus, 1758)	European badger	LC	S	11	4
*Mustela erminea* (Linnaeus, 1758)	Stoat	NT	S/G	67	29
*Mustela nivalis* (Linnaeus, 1766)	Common weasel	NT	S/G	5	3
*Mustela putorius* (Linnaeus, 1758)	European polecat	NT	S/G	9	5
*Neovison vison* (Schreber, 1777)	American mink	NA	S	34	9

*Note*: Study area codes: O = Overby Lyng, S = Strødam, and G = Gribskov.

**TABLE 4:** Independent seasonal trapping rates of stoat and weasel in the study areas of Strødam and Gribskov.

Season	Strødam, seasonal trapping rates		Gribskov, seasonal trapping rates	
	Stoat	Weasel	Stoat	Weasel
Autumn 2021	0.12	0.04	ND	ND
Winter 2021–2022	0.40	NO	ND	ND
Spring 2022	NO	0.04	ND	ND
Summer 2022	NO	NO	ND	ND
Autumn 2022	ND	ND	0.71	0.04
Winter 2022–2023	ND	ND	NO	NO

Abbreviations: ND, not deployed; NO, not observed.

Weasel was captured in two Double‐Mostelas in Strødam and one in Gribskov, while stoat was captured in three Double‐Mostelas in Strødam and one in Gribskov. Neither of the species was captured in Overby Lyng. Naive occupancy varied seasonally and in the different study areas (Table 5). Model estimated detection probability for stoat was 0.09 (95% CI 0.04–0.17) and occupancy probability was 0.04 (95% CI 0.01–0.21) during autumn in Gribskov (Table 5). During winter in Strødam, detection probability was 0.04 (95% CI 0.02–0.09) and occupancy probability was 0.07 (95% CI 0.02–0.24) in Strødam (Table 5). Due to low number of captures, we were unable to estimate detection and occupancy probabilities for stoats in autumn in Strødam and for weasels in both study areas. We were unable to run occupancy models using site covariates due to the low number of sites where stoats were captured.

**TABLE 5:** Seasonal naive occupancy, detection probability, and occupancy probability of stoats and weasels in Strødam and Gribskov.

Study area	Species	Season	Naïve occupancy	Detection probability estimate (*p*)	Occupancy probability estimate (Ψ)
Strødam	Stoat	Autumn 2021	0.10 (3)	—	—
		Winter 2021–2022	0.07 (2)	.04 (.02–.09)	.07 (.02–.24)
	Weasel	Autumn 2021	0.03 (1)	—	—
		Spring 2022	0.03 (1)	—	—
Gribskov	Stoat	Autumn 2022	0.04 (1)	.09 (.04–.17)	.04 (.01–.21)
	Weasel	Autumn 2022	0.04 (1)	—	—

*Note*: For naive occupancy, the number of Double‐Mostelas visited is given in parentheses. For detection probability (*p*) and occupancy probability (Ψ), the 95% confidence interval is given in parentheses. Due to low number of captures, model could not run and estimate detection probability and occupancy probability in all seasons. Only seasons in which stoats and weasels were detected were included.

Both stoats and weasels were captured before and during the use of scent‐based lures in the Double‐Mostelas in Strødam. During the treatment with the lure Weasel Supreme, stoat was captured in one of the Double‐Mostelas (Figure S1), where stoat had also been captured prior to the lure treatments. During the same treatment, we captured weasel in a Double‐Mostela where it had not been captured before treatments.

### 3.2 External cameras in Gribskov

A total of 494 captures were obtained during 1130 camera trap days of the external camera traps facing the Double‐Mostelas in Gribskov. Cameras operated for different time lengths, and the range of active deployment spanned from 5 to 111 days. Of the 494 photos, 197 were animal captures (including humans) and the rest were either blanks or photos taken during setup and pickup. The most common species recorded was the European roe deer (*Capreolus capreolus*) with 42 total captures (Table S3). There were no captures of stoats and weasels on the external cameras.

### 3.3 Identifying individuals

**FIGURE 6:**
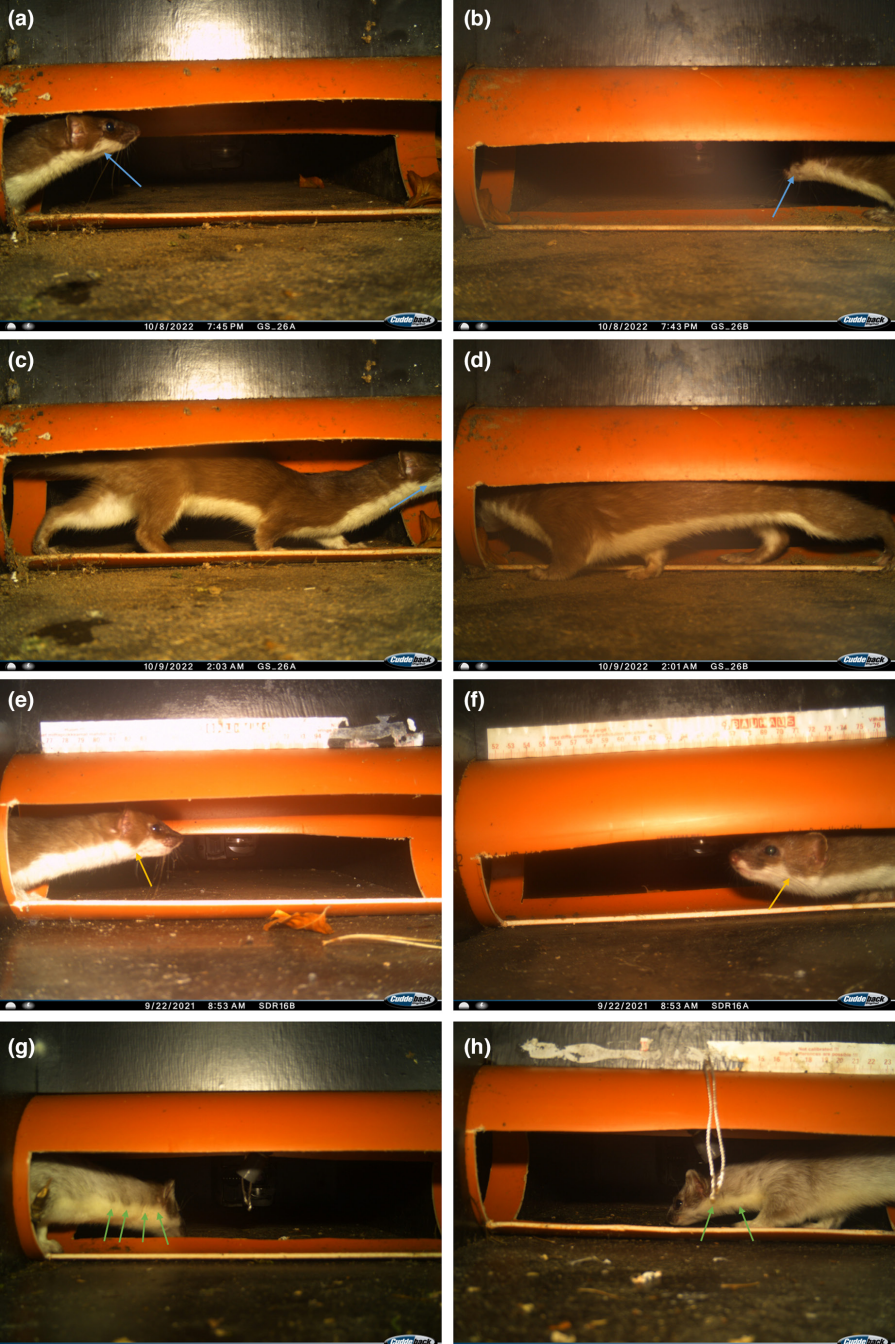
Doubled‐sided captures of stoat in Double‐Mostelas in Gribskov on different dates and times (a–d), stoat captured in SDR16 in Strødam (e,f), and in SDR30 in Strødam (g,h). Photos opposite from each other show the corresponding photo of the stoats from both cameras within the Double‐Mostela (a,b, c,d, e,f, and g,h). The stoat on a‐d has distinct brown spot in the white fur of the cheek indicated by the blue arrows (a + c). This distinct feature might be used for identification of individual stoats, and we may assume that this is in fact the same individual coming back to the Double‐Mostela multiple times. The distinct spot from a‐d is missing on the stoat on e and g. The triangular brown spot around the ear (indicated by yellow arrow in e) seems to be sharper and extend further than the corresponding spot on the stoats on a–d. The stoat in photo g has four distinct indentations on the neck (indicated by green arrows), which are missing in the other stoats.

We identified distinct features in the fur coat of the stoats we captured in both Strødam and Gribskov. In Gribskov, the Double‐Mostela GS26 had the only captures of stoats. The stoats captured all had a distinct brown spot in the white fur of the cheek (indicated by blue arrows in Figure 6a + c), and this spot was not present on stoats captured in Strødam (Figure 6e + g). One stoat had a more sharply outlined triangular brown area around the ear (indicated by the yellow arrow in Figure 6e), and this spot extends further down than the corresponding spot on the stoats captured in Gribskov (Figure 6a–d). The stoats captured in Strødam, SDR30, had four distinct indentations on the neck (indicated by green arrows in Figure 6g), which were lacking in all the other stoats (Figure 6a–f). This suggests that only a single individual stoat was captured in the same Double‐Mostela in Gribskov (Figure 6a–d) and that two individuals were captured in SDR16 and SDR30 in Strødam (Figure 6e–h). In all events, we identified potential features for identification of individuals. Going over captures from both cameras revealed that in 81%–93% of individual events, it was necessary to consult the opposite camera of the Double‐Mostela to identify features on both sides of the animal.

### 3.4 Test of lure

We obtained a total of 2940 photos during 1134 camera trap days when testing the different scent‐based lures in Strødam. Of these, 1791 were captures of animals, and the rest were blanks or photos taken during setup and pickup. When filtering for 60‐s independence, we obtained 413 independent animal captures. The most common species was the European fallow deer (*Dama dama*) with 1260 total captures (Table S4). The treatment with fewest independent animal captures was the control (*n* = 26) and the most captures were the lure Mega Musk with 71 independent animal captures. We performed a Kruskal–Wallis *H* test and found no significant difference between the type of lure used and number of animal captures (*p* = .7577). None of the small mustelids were captured during the deployment period. European pine marten was captured at site L03 4 days after the application of Weasel Supreme. European badger was captured at L06 during the control treatment. Multiple other non‐mustelid species showed an interest in the setup (Figures S2 and S3).

## 4 DISCUSSION

This study is the first test of the Double‐Mostela camera trapping device designed for capturing and individually identifying stoats and weasels. We captured both stoats and weasels on camera traps using this method but with very low seasonal capture rates, especially for weasels. We captured no stoat or weasel on the external cameras, and in Gribskov, the cameras deployed used xenon flash which could potentially result in missed captures of the fast‐moving species. In their study, using Mostela, Mos and Hofmeester (2020) found trapping rates of 19.9 and 8.6 weasels per 100 camera trap days during spring and summer in 2 consecutive years, and in another study from England, capture rates of 1.4 weasel per 100 camera trap days were found during spring and summer as well (Croose & Carter, 2019). These capture rates for weasels are much higher than our capture rates of 0.04 (autumn) and 0.04 (spring) weasels per 100 camera trap days in Strødam and 0.04 (autumn) in Gribskov. We found 0.12 and 0.40 stoats per 100 camera trap days in Strødam during autumn and winter, respectively, and 0.71 stoats per 100 camera trap days in Gribskov during autumn. Mos and Hofmeester (2020) did not find any stoat in their study in the Netherlands, and in England, Croose and Carter (2019) found 0.1 stoat per 100 camera trap days.

In contrast to what Croose and Carter (2019) found, we have a higher detection rate of stoats than weasels. It has been shown that stoats in particular can develop “trap‐shyness” and will avoid traps and tunnels (Brown, 2001; King et al., 2009) and they will enter traps less frequently if there is a high abundance of prey (King & White, 2004). We are potentially observing that weasels are trap shy because we observe more stoats and plenty of prey in our study areas. However, considering the hunting strategy of entering holes applied by weasels (King & Powell, 2007), something else may explain the higher trapping rate of stoats. We may capture more stoats than weasels based on different habitat preferences of the species, or potentially, the stoat is the dominant species in the areas surveyed, which could explain the lower trapping rate of weasels compared to stoats. However, further research would be needed to investigate this. Similarly, we are potentially observing the same phenomenon for weasels as Croose and Carter (2019) did for stoats, where stoat was observed in study areas but not captured in the Mostela. It was shown in a study on the Irish stoats (*M. e. hibernica*) with deployment of external cameras facing the Mostela that stoats were observed at four different sites but only entered the Mostela at two sites (Croose et al., 2021). They found 1.5 stoats per 100 camera trap days inside the Mostela, and 2.1 stoats per 100 camera trap days on the external cameras facing the Mostela (Croose et al., 2021). Compared to other studies, we have an intermediate trapping rate of stoats during the autumn deployment in Gribskov and we did not capture any stoats on the external cameras in Gribskov. However, a Danish study using traditional camera trap setup with cameras facing streams and stream banks in western Zealand found capture rates of 0.2 stoat and 0.02 weasel per 100 camera trap days (Holm et al., 2022). This trapping rate is lower than what we found using the Double‐Mostelas, indicating that the low capture rates in the Double‐Mostela are not caused by stoat and weasel being unwilling to enter the boxes.

Due to the low number of captures, we were not able to make density estimates. We were able to get model estimates of detection probability and occupancy probability for stoat captures during autumn in Gribskov and winter in Strødam. In both cases, the occupancy probability estimate was similar to the naive occupancy. We found a higher detection probability in autumn compared to winter. We did not capture any stoat or weasel during summer, where detection probabilities have otherwise been found to be highest (Hofmeester et al., 2024; Jedrzejewski et al., 1995).

We found weasels in areas characterized by grassy undergrowth and proximity to water. This resembles a large Polish study that found that weasel will be found in most habitats within or adjoining a forested area (Jedrzejewska & Jedrzejewski, 1998). However, both weasels and stoats are also known to use hedgerows, stone walls, and natural corridors in open natural areas and on farmland (King & Powell, 2007). These are the habitat types that are the primary focus of the studies with higher capture rates and detection probabilities of weasels using the Mostela (Croose & Carter, 2019; Mos & Hofmeester, 2020). The sites where we captured stoats seem to be slightly more forested than where we found weasels, but still close to open, grassy areas. However, these results must be interpreted with care considering our low capture rates and the low naive occupancies. Stoats found in the Polish study much preferred open, sedge marshes along rivers compared to forested riverplains (Jedrzejewska & Jedrzejewski, 1998).

All of this begs the question as to why we do not capture more stoats and weasels considering the length of deployment of cameras and the habitats covered. Of course, there can be an issue with a suboptimal setup and placement of the Double‐Mostelas. We deployed Double‐Mostelas primarily in forested areas or in areas heavily influenced by human activity, whereas other studies suggest that weasels and stoats will be found in the open land characterized by hedgerows and other linear features in the landscape (Mos & Hofmeester, 2020; Zub et al., 2008). However, in Denmark, hedgerows and other covered linear features in the open landscape are extremely rare due to the landscape transformation for intensive agriculture. It has been suggested that weasels may primarily use woodland in winter (King, 1975), which may explain the lack of observations during summer in our forested study areas. Weasel populations have been shown to fluctuate over the year, and to closely follow that of their prey (Jedrzejewski et al., 1995; Zub et al., 2008). Similarly, stoats have been found to change their habitat use and ranges over the years (Erlinge & Sandell, 1986). Therefore, we may have placed Double‐Mostelas in habitats less optimal for detecting stoats and weasels, and deployment periods in Overby Lyng and Gribskov may have been outside of the periods with highest detection probabilities.

However, taking into account the length of deployment in Strødam, the low trapping rates of stoat and weasel found in another part of Denmark by Holm et al. (2022), and the fact that we did not get any captures on external cameras in Gribskov points to something more being the cause of the low captures. From the 1950s, the yearly yield from hunting stoats declined from 6000–7000 to ~2000 in 1982 when the stoats became protected in Denmark (Jensen & Jensen, 1973). The decrease in stoats could not only be ascribed to a lower hunting effort but also a decline in population size (Jensen & Jensen, 1973). In 1970/1971, weasel sightings and kills in stoat traps were registered by hunters, and the general picture given was that the weasel was widespread throughout Denmark but, like the stoat, the population seemed to be declining (Jensen & Jensen, 1973). Similar declines in stoat and weasel capture have been observed using various methods in Finland (Hellstedt et al., 2006), Spain (Torre et al., 2018), Switzerland (Akdesir et al., 2018), and the United States (Jachowski et al., 2021). Several reasons can be proposed as to why the populations of stoats and weasels have been declining.

The last 200 years have seen dramatic changes to the landscape in Denmark (Sand‐Jensen & Schou, 2019), and with 63.7% of the land used for agriculture and just 2.3% strictly protected nature areas (Levin, 2019), species are experiencing highly fragmented habitats. In Denmark, we have specifically seen a change to larger fields and thus a reduction in hedgerows and natural corridors (Sand‐Jensen & Schou, 2019). Stone walls have also been removed in many places or changed in ways that reduce their attraction to wildlife (Sand‐Jensen & Schou, 2019). A lot of these changes have removed habitats that weasels and stoats are otherwise known to use (Macdonald et al., 2004; Zub et al., 2008), thus not only removing places for them to live but also decreasing the possibility for them to disperse and meet potential mates.

Despite being specialized predators, stoats and weasels are also highly in danger of predation themselves. They are potential prey for the introduced mink, foxes (*Vulpes vulpes*), and birds of prey (King & Powell, 2007). In Denmark, there has been a rise in birds of prey over the years, and currently, 8 of 13 raptors breeding in Denmark are increasing in population size (DOF, 2020). It has been shown that both stoats and weasels are killed by free‐roaming, domestic cats (Elmeros et al., 2007a, 2007b). There are an estimated 730,000 domestic (Lund & Sandøe, 2021) and 90,000 unowned (Nielsen et al., 2022) cats in Denmark, and as many of them roam freely, there are concerns that these might influence population densities of their prey (Doherty et al., 2016; Loss et al., 2013). The risk of predation is thought to be the main driver for weasels and stoats living in far northern areas to change into a white winter coat (King & Powell, 2007). In Denmark, the stoat is the only mammal that changes into a white winter fur, whereas the weasel does not change. Over the last 50 years, we have seen marked changes in the amount of snowfall and days with snow cover in Denmark (de Vries et al., 2014), and this might be a problem for the white stoats in wintertime. Having a white fur when there is no snow cover might prove to be a great disadvantage for the stoat, as it is more easily spotted by a predator against a dark background.

Apart from being at risk of predation, stoats and weasels might also experience dangers in hunting poisoned prey. A Danish study tested opportunistically collected stoat and weasel carcasses and found that anticoagulant rodenticides were found in 97% of weasels and 95% of stoats (Elmeros et al., 2011). Concentrations of rodenticides are higher in stoats and weasels with unknown causes of death compared to the ones with visual physical injury (Elmeros et al., 2011). This seems to indicate that the extensive use of rodenticides to kill rats and mice is affecting stoats and weasels, likely if they eat prey with rodenticides in their bodies.

We tested eight different scent‐based lures and one control treatment during this study, and we did not observe any effect of the use of lures in attracting mustelids. However, due to the limited captures of mustelids and therefore not being able to statistically test for an effect, this has to be interpreted with caution. The use of similar brands of American scent‐based lures was found to increase detection rates of European pine marten in Switzerland (Burki et al., 2010), but had no effect on stoat and weasel captures in England (Croose & Carter, 2019). We found no significant differences between treatments potentially due to the low capture rates. Captures of the small mustelids in the Double‐Mostela and the European pine marten on external cameras during the treatments all occurred using the lure Reuwsaat's Weasel Supreme (Figures S1 and S2c). Again, this must be interpreted with caution as the observations of mustelids during this treatment may have simply been coincidences. We observed European pine marten, red fox, and European badger showing an interest in the stake with the lure attached, but deer were also recorded investigating it (Figures S2 and S3). The control treatment has the least number of animal captures (*n* = 26), and the lures might, in fact, attract animals in general, and not specifically mustelids. Since we captured both weasel and stoat prior to the application of lure inside the Double‐Mostelas we deem it unnecessary to use these scent‐based lures for attracting mustelids in future efforts in Denmark.

For weasels, captures were so few that we could not look at distinguishing individuals; however, with stoats, we have identified some distinct spots and patterns in the fur that might be used for identifying individuals (Figure 6). Even though some of the events of the stoat in the Double‐Mostela showed both sides on a single camera, the ability to distinguish specific features in individuals is greatly increased by using the Double‐Mostela. The potential of having usable photos for identification from both sides of the animal increases the chance of distinguishing individuals and comparing them with other captures. The benefit of more solid identification of individuals, possible with the Double‐Mostela, makes it preferable to use, when monitoring specifically for stoats and weasels, even considering the increased cost of the extra camera in each Double‐Mostela.

In this study, we aimed to make density estimates of weasels and stoats based on identification of individuals at three different locations in northern Zealand, Denmark. However, due to low capture rates, we were not able to do so. The camera trapping method proved capable of capturing both weasel and stoat and is worth additional investigation. To our knowledge, this is the first study that has identified distinguishing features in the fur of stoats. With the low capture rates found in this study, it might be beneficial to test the Double‐Mostela on other locations in Denmark as we cannot know if our results are representative of the country or not. Due to the effort needed when using lures to attract mustelids and the results we gained from using them, we suggest not using commercial scent‐based lures for future studies in Denmark. With higher number of captures of stoats and weasels, further development of the individual identification could be applied along with capture–recapture models to obtain density estimates.

## AUTHOR CONTRIBUTIONS

**Sofie Nørgaard Konradsen:** Conceptualization (supporting); data curation (lead); formal analysis (lead); investigation (equal); methodology (equal); project administration (supporting); visualization (lead); writing – original draft (lead). **Linnea Worsøe Havmøller:** Conceptualization (equal); data curation (supporting); funding acquisition (supporting); investigation (equal); methodology (supporting); supervision (equal); writing – review and editing (equal). **Charlotte Krag:** Data curation (equal); investigation (equal); methodology (supporting); writing – review and editing (equal). **Peter Rask Møller:** Supervision (equal); writing – review and editing (equal). **Rasmus Worsøe Havmøller:** Conceptualization (equal); data curation (equal); formal analysis (supporting); funding acquisition (lead); investigation (equal); methodology (lead); project administration (lead); resources (lead); supervision (lead); validation (equal); visualization (supporting); writing – original draft (equal); writing – review and editing (equal).

## CONFLICT OF INTEREST STATEMENT

We have no conflicts of interest to declare.

## Supporting information


Appendix S1.

Appendix S2.

Appendix S3.

Appendix S4.

Appendix S5.

Appendix S6.

Appendix S7.

Appendix S8.

## Data Availability

The data that support the findings of this study will be submitted as Supporting Information. Data can be found as Supporting Information as “DataGribskovExternal.csv,” “DataLureElusiveMustelids.csv,” and “DataElusiveMustelids.csv.”. The data is available on GBIF and can be accessed at: https://doi.org/10.15468/effnsb.
