# Supplementary material for: Elusive mustelids—18 months in the search of near‐threatened stoat (Mustela erminea) and weasel (M. nivalis) reveals low captures
Source: Ecol Evol. 2024 May 1;14(5):e11374. doi: 10.1002/ece3.11374 (PMC11063614; doi:10.1002/ece3.11374)
Supplement: Supplementary file 8 — Appendix S8. [file ECE3-14-e11374-s001.docx]

## Appendix

Table S1: Overview of the American scent-based lures tested during the end of the deployment period of the Double-Mostelas in Strødam.

| Period | Start date | End date | Days | Lure type |
| --- | --- | --- | --- | --- |
| 1 | 2022-03-17 | 2022-04-19 | 33 | Weasel Supreme (Reuwsaat’s) |
| 2 | 2022-04-19 | 2022-05-09 | 21 | Marten Super all call (Lenon’s) |
| 3 | 2022-05-09 | 2022-06-08 | 29 | Gusto, Long Distance Call Lure, Predator Lure (Caven’s) |
| 4 | 2022-06-08 | 2022-09-20 | 102 | Weasel Super all call (Lenon’s) |

Table S2: Raw and 60 second independent captures of all species found in the Double-Mostelas at the three study areas.

| **Species** | **Total captures, Overby Lyng** | **Independent captures, Overby Lyng** | **Total captures, Strødam** | **Independent captures, Strødam** | **Total captures, Gribskov** | **Independent captures, Gribskov** |
| --- | --- | --- | --- | --- | --- | --- |
| *Anas platyrhynchos* |  |  | 3 | 1 |  |  |
| *Anguis fragilis* | 1 | 1 |  |  |  |  |
| *Apodemus flavicollis* | 107 | 60 | 10198 | 5401 | 8199 | 3914 |
| *Apodemus sylvaticus* | 8 | 4 | 876 | 476 | 402 | 192 |
| *Apodemus sp.* |  |  | 35 | 22 | 657 | 370 |
| *Arvicola amphibius* |  |  | 80 | 35 | 1 | 1 |
| *Bos taurus* |  |  | 20 | 8 |  |  |
| *Bufo bufo* |  |  | 2 | 2 |  |  |
| *Canis lupus familiaris* |  |  | 7 | 3 | 3 | 2 |
| *Cyanistes caeruleus* |  |  | 9 | 8 | 9 | 8 |
| *Equus ferus* |  |  | 18 | 7 |  |  |
| *Erithacus rubecula* |  |  | 897 | 459 | 27 | 18 |
| *Felis catus* |  |  | 10 | 4 | 5 | 3 |
| *Lacerta agilis* |  |  | 3 | 3 |  |  |
| *Martes foina* | 4 | 2 |  |  |  |  |
| *Martes martes* |  |  |  |  | 2 | 1 |
| *Meles meles* |  |  | 11 | 4 |  |  |
| *Micromys minutus* |  |  | 54 | 23 | 92 | 46 |
| *Microtus agrestis* | 90 | 43 | 10284 | 4536 | 735 | 387 |
| *Mustela erminea* |  |  | 30 | 13 | 37 | 16 |
| *Mustela nivalis* |  |  | 3 | 2 | 2 | 1 |
| *Mustela putorius* |  |  | 4 | 2 | 5 | 3 |
| *Myodes glareolus* | 38 | 17 | 29420 | 14234 | 20792 | 8889 |
| *Natrix natrix* |  |  | 5 | 5 |  |  |
| *Neomys fodiens* |  |  | 518 | 215 | 129 | 70 |
| *Neovison vison* |  |  | 34 | 9 |  |  |
| *Parus major* |  |  | 37 | 32 | 17 | 14 |
| *Passer domesticus* |  |  | 1 | 1 |  |  |
| *Phoenicurus phoenicurus* |  |  | 7 | 5 |  |  |
| *Plecotus auritus* |  |  | 3 | 1 | 2 | 1 |
| *Rattus norwegicus* |  |  | 15 | 5 |  |  |
| *Regulus regulus* |  |  |  |  | 2 | 2 |
| *Sciurus vulgaris* |  |  | 108 | 62 | 163 | 79 |
| *Sorex araneus* | 38 | 21 | 8173 | 4485 | 1014 | 526 |
| *Sorex minutus* | 40 | 30 | 3873 | 2159 | 781 | 443 |
| *Sorex sp.* |  |  | 384 | 248 | 150 | 91 |
| *Tadorna tadorna* |  |  | 1 | 1 |  |  |
| *Troglodytes troglodytes* |  |  | 254 | 189 | 58 | 48 |
| *Turdus merula* | 16 | 8 | 20 | 15 |  |  |
| *Vipera berus* |  |  | 7 | 7 |  |  |
| *Vulpes vulpes* |  |  | 3 | 3 |  |  |
| *Zootoca berus* |  |  | 1 | 1 |  |  |
| **Total** | 342 | 186 | 65408 | 32686 | 33284 | 15125 |

Table S3: Species inventory of external cameras in Gribskov with total and independent 60 second captures. *Humans captured in the cameras and annotated here are guests in the forest. All photos containing people have been treated confidentially and deleted post-analysis as is required by the GDPR legislation.

| Order/  family | Scientific name | Vernacular name | Total captures | Independent captures |
| --- | --- | --- | --- | --- |
| **Rodentia**  Muridae  Sciuridae | *Apodemus flavicollis* (Melchior, 1834)  *Sciurus vulgaris* (Linnaeus, 1758) | Yellow-necked mouse  Red squirrel | 7  18 | 7  18 |
| **Carnivora**  Canidae  Canidae  Felidae  Mustelidae  Mustelidae  Mustelidae | *Canis lupus familiaris* (Linnaeus, 1758)  *Vulpes vulpes* (Linnaeus, 1758)  *Felis catus* (Linnaeus, 1758)  *Martes martes* (Linnaeus, 1758)  *Meles meles* (Linnaeus, 1758)  *Mustela putorius* (Linnaeus, 1758) | Domestic dog  Red fox  Domestic cat  European pine marten  European badger  European polecat | 5  15  20  3  2  4 | 3  15  19  3  2  4 |
| **Artiodactyla**  Cervidae  Cervidae  Cervidae | *Capreolus capreolus* (Linnaeus, 1758)  *Cervus elaphus* (Linnaeus, 1758)  *Dama dama* (Linnaeus, 1758) | European roe deer  Red deer  European fallow deer | 42  1  32 | 36  1  27 |
| **Columbiformes**  Columbidae | *Columba palumbus* (Linnaeus, 1758) | Common wood pigeon | 7 | 6 |
| **Primates**  Hominidae | *Homo sapiens* (Linnaeus, 1758) | Human* | 39 | 30 |
| **Passeriformes**  Paridae  Paridae | *Cyanistes caeruleus* (Linnaeus, 1758)  *Parus major* (Linnaeus, 1758) | Blue tit  Great tit | 1  1 | 1  1 |

Table S4: Species inventory of external cameras in Strødam during test of scent-based lures with total and independent 60 second captures. *Humans captured in the cameras and annotated here are guests in the forest. All photos containing people have been treated confidentially and deleted post-analysis as is required by the GDPR legislation.

| Order/  family | Scientific name | Vernacular name | Total captures | Independent captures |
| --- | --- | --- | --- | --- |
| **Rodentia**  Sciuridae | *Sciurus vulgaris* (Linnaeus, 1758) | Red squirrel | 18 | 6 |
| **Carnivora**  Canidae  Felidae  Mustelidae  Mustelidae | *Vulpes vulpes* (Linnaeus, 1758)  *Felis catus* (Linnaeus, 1758)  *Martes martes* (Linnaeus, 1758)  *Meles meles* (Linnaeus, 1758) | Red fox  Domestic cat  European pine marten  European badger | 96  24  3  3 | 32  8  1  1 |
| **Artiodactyla**  Cervidae  Cervidae  Cervidae | *Capreolus capreolus* (Linnaeus, 1758)  *Cervus elaphus* (Linnaeus, 1758)  *Dama dama* (Linnaeus, 1758) | European roe deer  Red deer  European fallow deer | 138  3  1260 | 34  1  279 |
| **Columbiformes**  Columbidae | *Columba palumbus* (Linnaeus, 1758) | Common wood pigeon | 39 | 11 |
| **Primates**  Hominidae | *Homo sapiens* (Linnaeus, 1758) | Human* | 27 | 8 |
| **Passeriformes**  Muscicapidae  Paridae  Paridae | *Erithacus rubecula* (Linnaeus, 1758)  *Cyanistes caeruleus* (Linnaeus, 1758)  *Parus major* (Linnaeus, 1758) | European robin  Blue tit  Great tit | 3  3  3 | 1  1  1 |
|  |  |  |  |  |
| **Domesticated animals** |  |  |  |  |
| **Artiodactyla**  Bovidae | *Ovis aries* (Linnaeus, 1758) | Sheep | 174 | 29 |


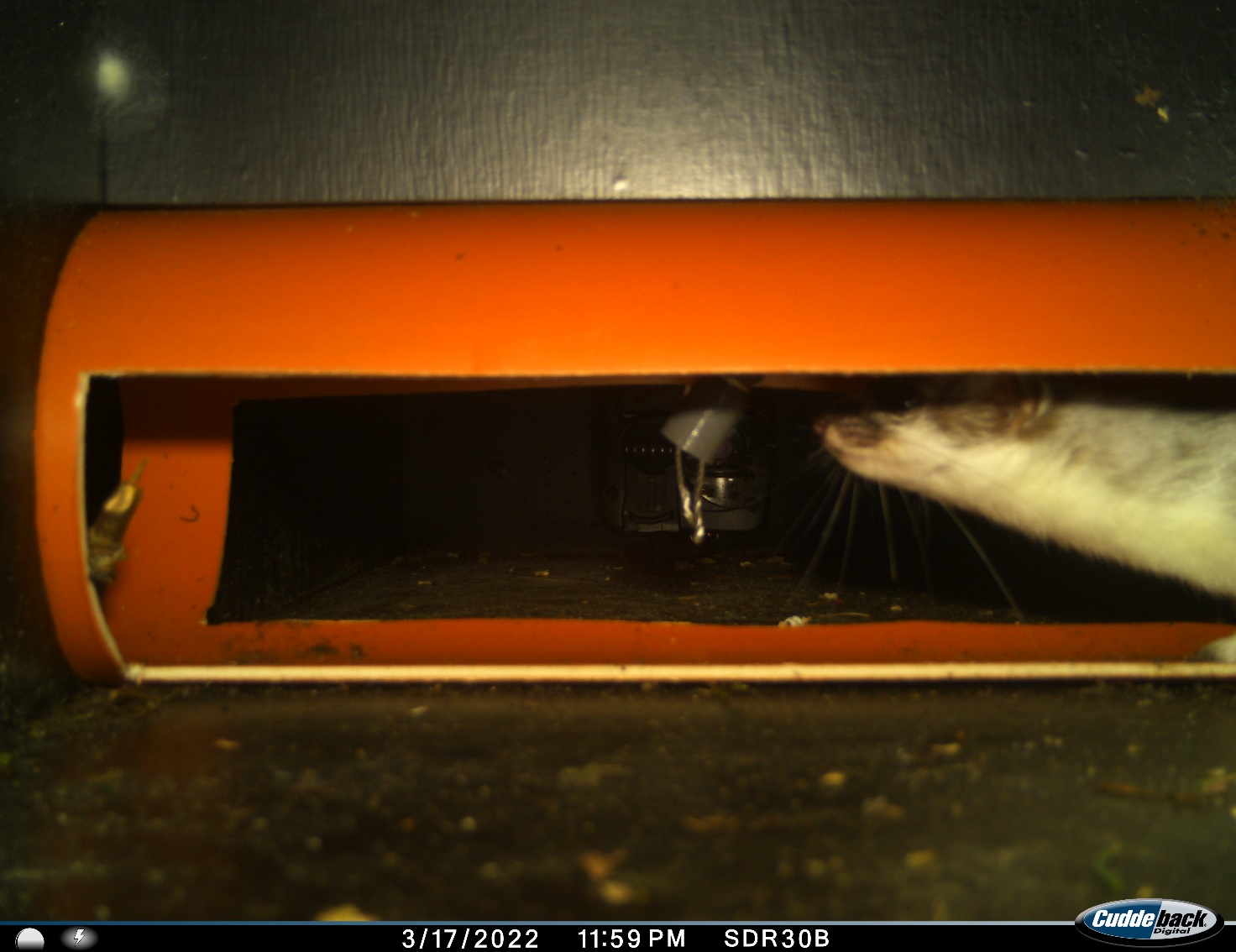


Figure S1: Stoat (*Mustela erminae*) in SDR30 on 2022-03-17. The scent-based lure, Weasel Supreme, had been applied earlier on the same day. The stoat can be seen smelling the container with the lure.


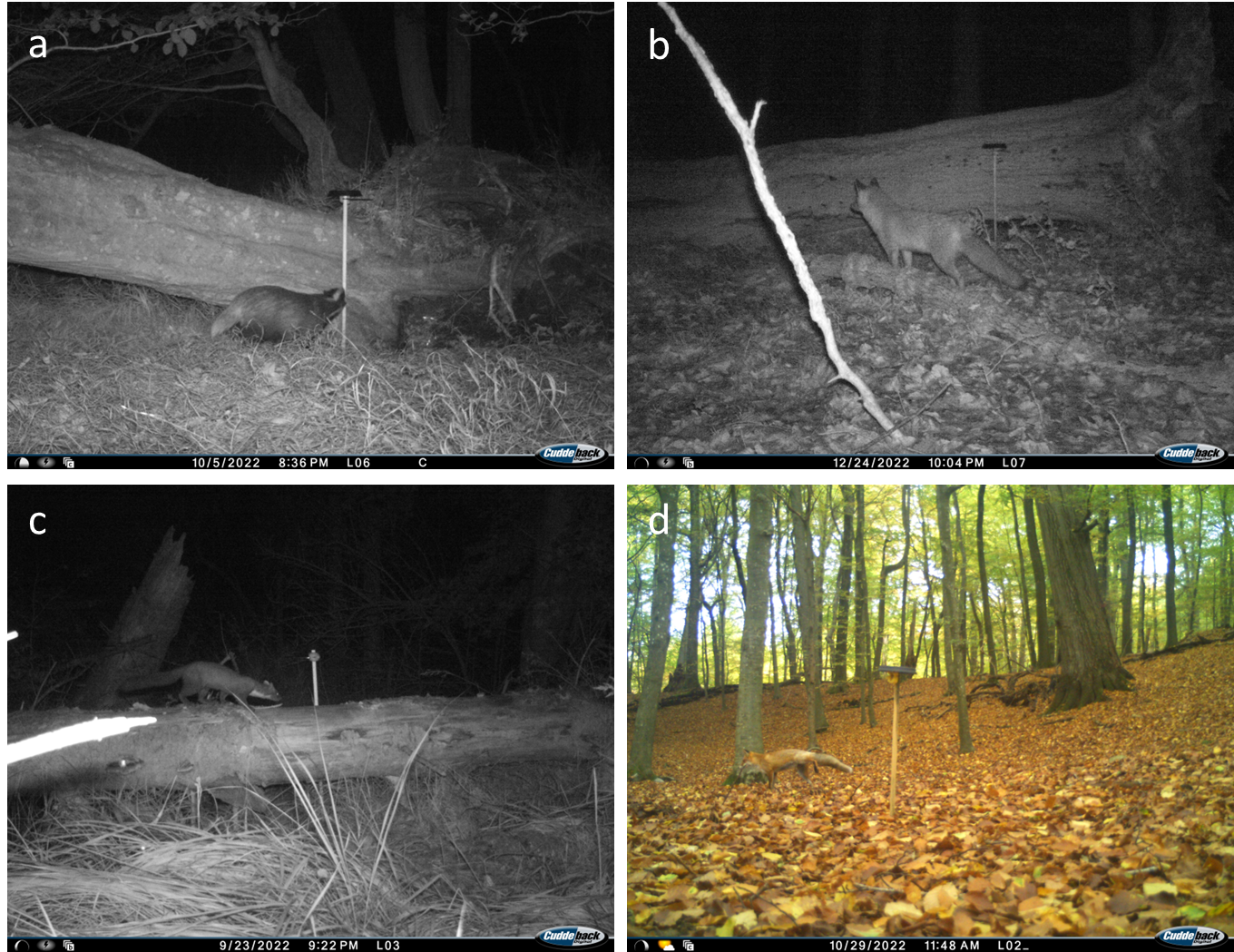


Figure S2: a) European badger (*Meles meles*) during control treatment, b) red fox (*Vulpes vulpes*) during Mega Musk treatment, c) European pine marten (*Martes martes*) during Weasel Supreme treatment and d) red fox during Mega Musk treatment


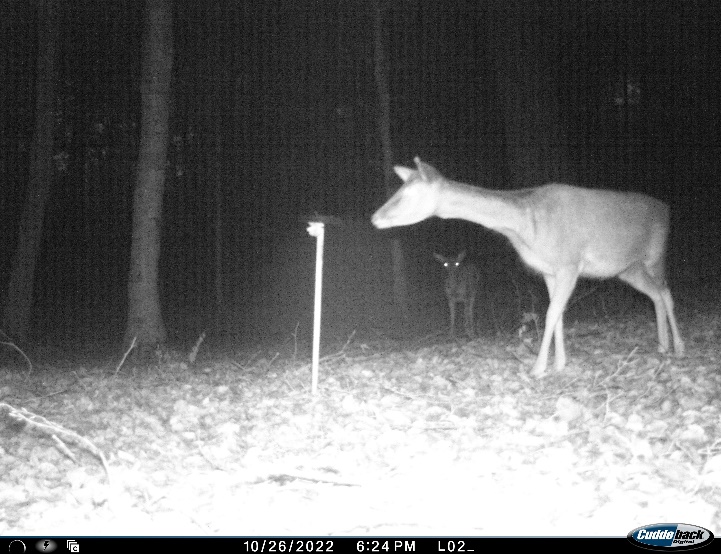

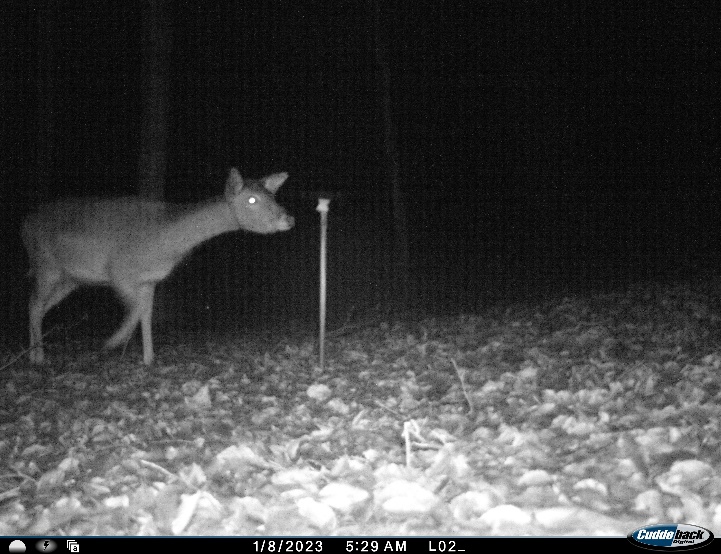

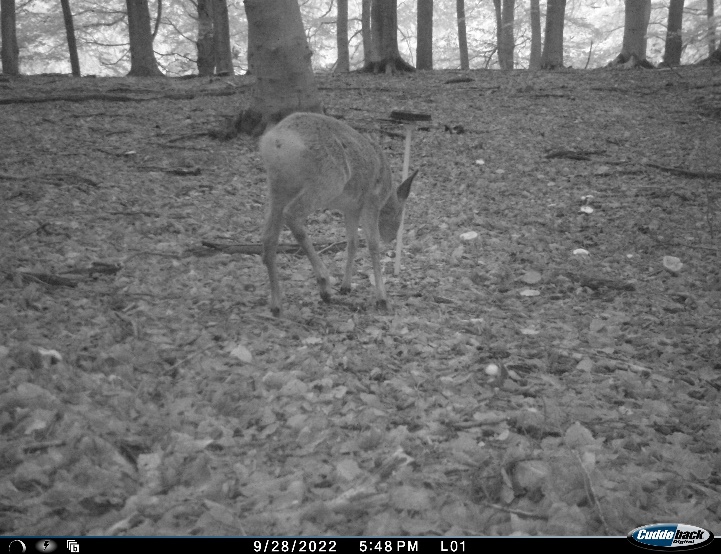

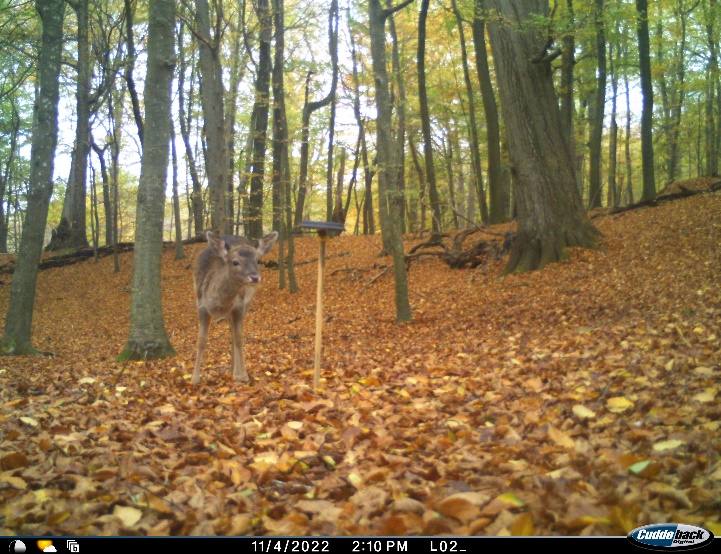


a

d

c

b

Figure S3: European fallow deer (*Dama dama*) during a) Mega Musk treatment, b) Marten Super All Call treatment and c) Weasel Super All Call treatment, and d) roe deer (*Capreolus capreolus*) during Shellfish Oil treatment.
